# Effects of Hyaluronic Acid (HA) and Platelet-Rich Plasma (PRP) on Mandibular Mobility in Temporomandibular Joint Disorders: A Controlled Clinical Trial

**DOI:** 10.3390/biom14101216

**Published:** 2024-09-26

**Authors:** Maciej Chęciński, Dariusz Chlubek, Maciej Sikora

**Affiliations:** 1 Department of Oral Surgery, Preventive Medicine Center, Komorowskiego 12, 30-106 Kraków, Poland; maciej@checinscy.pl; 2 Department of Biochemistry and Medical Chemistry, Pomeranian Medical University, Powstańców Wielkopolskich 72, 70-111 Szczecin, Poland; sikora-maciej@wp.pl; 3 Department of Maxillofacial Surgery, Hospital of the Ministry of Interior, Wojska Polskiego 51, 25-375 Kielce, Poland

**Keywords:** temporomandibular joint, temporomandibular disorders, intra-articular injections, hyaluronic acid, platelet-rich plasma

## Abstract

Hyaluronic acid (HA) is a glycosaminoglycan composed of D-glucuronic acid and N-acetylglucosamine with an up-to-several-million-Daltons chain-length responsible for the lubricating properties of the temporomandibular joint (TMJ) synovial fluid. Arthritis results in the predominance of HA degradation over synthesis leading to temporomandibular disorders (TMDs). TMD injection treatments are divided into HA supplementation and platelet-rich plasma (PRP) inflammation suppression. We questioned whether either approach lubricated the TMJ better and answered it in a two-arm equal-allocation trial with a non-concurrent active treatment control (two groups of 39 patients each). HA statistically significantly improved (*p* < 0.01) and PRP did not statistically significantly change (0.06 ≤ *p* ≤ 0.53) articular mobility compared to baselines in 128 TMJs. Statistically significant inter-group discrepancies were observed for abduction (MD = −4.05 mm; SE = 1.08; *p* = 0.00; *d* = −0.85) and protrusion (MD = −0.97 mm; SE = 0.43; *p* = 0.03; *d* = −0.51) but not for rightward (MD = −0.21; SE = 0.43; *p* = 0.63; *d* = −0.11) and leftward (MD = −0.30; SE = 0.42; *p* = 0.47; *d* = −0.16) movements. HA supplementation proved superior to PRP autografting in ad hoc TMJ lubrication and hence is more appropriate in hypomobile TMD cases of symptomatic treatment.

## 1. Introduction

Hyaluronic acid (HA) belongs to the group of glycosaminoglycans, which are natural structural elements of living organisms and can be characterized by biological activity [1]. From a molecular point of view, glycosaminoglycans are polysaccharides composed of repeating disaccharide units, i.e., uronic acid and amino sugar. In the case of HA, these are D-glucuronic acid and N-acetylglucosamine. They are alternately connected by β-(1-4)- and β-(1-3)-glycosidic bonds [2].

In vivo, HA most often takes the form of sodium hyaluronate [3]. It is synthesized on the inner side of the plasma membrane in the presence of hyaluronan synthase enzymes [2]. This process occurs in both eukaryotic cells and bacteria. Therefore, HA for pharmaceutical purposes is obtained from fermentation, mainly in *Streptococcus equi* bacterial cultures [4]. The constant synthesis of HA in tissues is balanced by degradation involving enzymes, namely hyaluronidases. The speed of the changes that occur affects the macroscopic differences between tissues. The half-life of HA in the skin is up to a day and in cartilage even 20 days [5,6].

HA is a collective term for chemical compounds with different polymer chain lengths. On this basis, basic types of HA are distinguished: low molecular weight (LMW-HA), medium molecular weight (MMW-HA), and high molecular weight (HMW-HA). Concerning commercially available injectables, their molecular weights are 500–1200, 1000–2900, and 6000 kDa, respectively [7]. The size of hyaluronic acid molecules affects its physical properties, which in turn determines its applications. In physiological conditions, synovial fluid contains HA weighing more than 3000 kDa [8]. In the case of inflammation, this value drops statistically significantly to a median of about 1500 kDa [8]. This indicates the predominance of degradation processes of polymer chains over their synthesis in arthritis and justifies the idea of HA supplementation with intra-articular injections as a treatment [9,10].

Another approach for maintaining the correct composition of synovial fluid is to stop the degradation processes resulting from inflammation. The first abnormalities in the cartilage tissue may result from mechanical damage and systemic diseases, stimulating the secretion of inflammatory mediators from chondrocytes [11]. Cytokines are of the greatest importance here, including interleukin 1 β (IL-1-β) and interleukin 6 (IL-6), tumor necrosis factor α (TNF-α), metalloproteinases (MMPs), and free oxygen radicals (ROS) [12]. Inflammation of cartilage tissue leads to impaired chondrocyte differentiation and the predominance of degenerative processes over regenerative ones [11]. This initiates inflammation of the adjacent bone tissue and synovial membrane [11].

Synovitis begins in the vicinity of the cartilage degeneration foci and later progresses to further areas. On the one hand, inflammatory mediators initiate synovium degeneration, and on the other hand, they enhance the secretion of other mediators also from this tissue [13,14]. In this way, biologically active substances originating from the synovial membrane negatively affect the metabolism of cartilage tissue. Overall, this provides a positive feedback loop. Full-blown TMJ arthritis is characterized by the presence in the synovial fluid of inflammatory mediators released from damaged cells of cartilage, bone, and synovial tissue [14].

The mandible is moved by two temporomandibular joints (TMJs). A single TMJ consists of the cartilage-covered articular surfaces on the anterior part of the mandibular fossa and the articular tubercle on the temporal bone, both articular disc surfaces, and the articular surface on the mandibular head [15]. Therefore, movements in a TMJ involve two functional joints, located above and below the articular disc [15,16,17]. During abduction, the mandibular head initially rotates relative to the underside of the articular disc, and then, the superior surface of the disc slides onto the articular tubercle [18,19,20].

**Figure 1:**
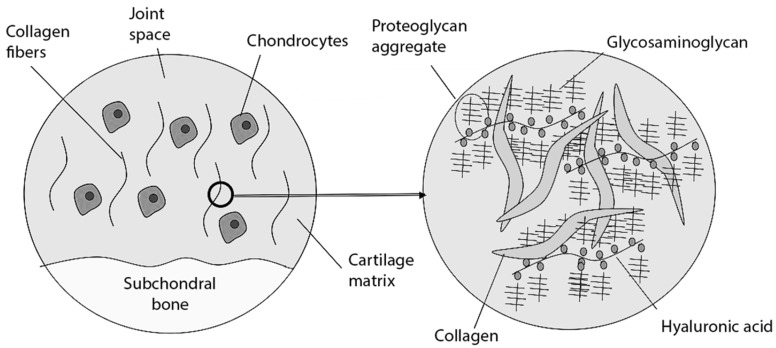
Articular surface. Adapted. Author: Mfigueiredo. License: CC BY-SA 3.0 (https://creativecommons.org/licenses/by-sa/3.0/) [29].

The articular surfaces are covered with cartilage, allowing them to slide smoothly against each other [21,22]. It comprises chondrocytes and an extracellular matrix (Figure 1) [21,22]. The latter consists of type II collagen, responsible for mechanical strength and elasticity, and HA, which forms complexes with proteoglycans [23,24]. These complexes retain water, which further improves the elasticity and shock-absorbing ability [23,25]. Physiologically, articular cartilage lacks blood vessels, lymphatic vessels, and nerves [26]. Its nutrition is mainly through diffusion from the subchondral bone and synovial fluid [27,28].

Synovial fluid fills an articular capsule surrounding the above structures [28,30]. The fluid (1) acts as a lubricant, reducing friction between joint surfaces; (2) acts as a shock absorber, protecting articular cartilage against mechanical damage; and (3) nourishes joint cartilage and helps maintain its elasticity [28,30]. The synovial fluid consists of hyaluronic acid (HA), water, proteins, and lipids [9]. It also contains enzymes, cytokines, and growth factors [31,32].

Internal derangement, autoimmune diseases, infections, and other factors trigger an inflammatory response [33,34]. Synovitis affects the composition of the capsular fluid, which are (1) decreased viscosity due to decreased HA production, (2) an increase in the number of inflammatory cells, (3) an increased concentration of pro-inflammatory cytokines, (4) an increase in the concentration of cartilage-damaging enzymes, (5) changes in the composition of proteoglycans and lipids, and (6) the retention of tissue degeneration products [31,33,34].

Temporomandibular disorders (TMDs) are a group of diseases that primarily present with pain and masticatory dysfunction [35,36]. Patients suffering from TMJ osteoarthritis, internal derangement, and degenerative joint disease complain of articular pain, limited mandibular mobility, and acoustic symptoms from the joint [35,36]. The prevalence of TMDs is, according to a meta-analysis, approximately 31% in adults worldwide, and approximately 11% in adolescents and children [37]. In the TMD group, the most common diagnosis is a displacement of the articular disc without blockage, which produces acoustic symptoms and may cause pain [36,37].

The progression of TMDs from TMJs involves increasing the frequency of clicking and cracking in the joints, transitioning from intermittent joint pain to chronic, and decreasing mobility of the mandible in all directions [35,38,39]. This contributes to mastication difficulties and, in severe cases, also spontaneous symptoms, resulting in a health-related quality of life decrease [38,40,41].

A TMD diagnosis is attempted to be specified through a medical interview, physical examination, and additional tests under the applicable classifications [35,36,42]. In particular, it is important to identify injuries, recurrent dislocation, and systemic diseases manifesting in the TMJ [35,36,43]. After excluding the above, an attempt is made to diagnose arthritis, disc displacement, or degenerative joint disease, although they may coexist [35,36,44]. The group of diseases discussed is of interest to (1) dentists, in particular specialists in dental prosthetics, orthodontics, dental surgery, and maxillofacial surgery, (2) physiotherapists, and (3) pain medicine physicians [45,46]. Unlike other joints, the TMJ is rarely considered an object of diagnosis and treatment by orthopedic specialists [47].

The various stages of degenerative TMJ disease involve progressive damage to the cartilage of the joint surfaces [21,22,48]. The priority is to remove the etiological factors of extra-articular overload resulting from excessive and incorrectly applied forces [49,50]. For this purpose, psychotherapy, physiotherapy, pharmacotherapy, and splint therapy are used [51,52,53]. Therapeutic actions on the joint itself may be aimed at delaying the development of degeneration or attempting to repair the damage [54,55].

The multitude of specialists involved in TMD treatment and the wide range of available therapeutic methods are evidence of the complexity of the health problem and the lack of simple solutions [46,56]. Natural matching of treatment to the disease is often impossible due to the lack of the possibility of a specific diagnosis or the coexistence of several disorders. In situations where causal action is impossible, symptomatic treatment is undertaken. Depending on the needs, attempts are made to relieve pain and improve jaw mobility [57,58]. Less invasive methods take precedence, which promotes physiotherapy and splint therapy [56,59,60]. For a lack of response or inconvenience of previous treatment, minimally invasive surgical techniques, such as joint cavity lavage (arthrocentesis) and intra-articular injections, are worth considering [10,61].

Among the numerous substances administered into the TMJ capsule, the best studied are hyaluronic acid, autologous platelet-rich plasma, and corticosteroids [46,62,63]. The latter group causes most of the observed injection therapy adverse effects [64]. As explained above, HA is a naturally occurring biopolymer, an important element of joint cartilage, and the main component of synovial fluid [9]. Direct intracavitary injections can supplement its deficiency resulting from chronic inflammation [10,48,58].

To regenerate damaged cartilage, attempts are made to reduce the concentration of inflammatory mediators and lytic enzymes and provide stem cells [54,55]. For this purpose, blood products and fat tissue autologous transplants are performed [55,63]. Various concentrates are centrifuged from peripheral venous blood, with the most literature regarding TMJ injection treatment involving platelet-rich plasma (PRP) [38,46,65]. Although experimental studies support the validity of PRP injections into the temporomandibular joint cavities, assessing such procedures’ ad hoc pro-mobility effectiveness is difficult due to various therapeutic protocols [66,67].

This study aims to compare the multidirectional mandibular mobility following the treatment of TMJ disorders with same-protocol intra-articular injections of PRP and HA. It is questioned whether there is direct clinical evidence of the superiority of PRP or HA in the injection treatment of TMJ disorders.

## 2. Materials and Methods

### 2.1. Registration

The study was conducted according to the guidelines of the Declaration of Helsinki and approved by the Bioethics Committee in Kielce at the Świętokrzyska Medical Chamber (2.3/2024; 18 July 2024). The study was registered in ClinicalTrials.gov (NCT06530745).

### 2.2. Trial Design

This study was designed as a two-arm equal-allocation clinical trial with a non-concurrent active treatment control. The study group consisted of consecutive patients receiving PRP injections into the TMJ cavities. The control group consisted of the same number of patients who received HA according to the same protocol. Controls were recruited consecutively, men and women separately, to maintain a consistent male-to-female ratio.

### 2.3. Participants

The main condition for recruitment was the diagnosis of TMJ pain by a specialist in orthodontics or dental prosthetics and, on this basis, a referral for injection treatment. TMJ pain was diagnosed under the International Classification of Orofacial Pain, 1st edition (ICOP 2020) guidelines [36]. Detailed eligibility criteria are presented in Table 1. The treatment was performed at the Maxillofacial Surgery Clinic in Kielce, Poland.

### 2.4. Interventions

The study group received PRP from peripheral venous blood of the elbow bend centrifuged at 160 revolutions per minute for 5 min. The control group received 2% (20 mg/mL) 2100 kDa HA. In both groups, 0.4 mL of the solution was injected per joint.

The active substance was administered via percutaneous injection into the TMJ cavity: (1) the injection point was located on the skin of the preauricular area according to the typical protocol; (2) the skin surface was disinfected; (3) the active substance was administered using a sterile 2 mL syringe with a sterile injection needle (various manufacturers); (4) post-operative recommendations were given (cooling the pre-auricular area, soft diet, reporting if complications were observed) [68]. No anesthesia or arthrocentesis was performed.

### 2.5. Outcomes

Self-authored questionnaires, including the medical history and physical examination, were prospectively designed. To assess outcomes, the following variables were collected: (1) range of maximum mandibular abduction; (2) range of anterior mobility of the mandible (protrusion); (3) extent of mandibular movement to the right; (4) extent of mandibular movement to the left. Mandibular mobility was assessed between the anthropometric points Incision superius (Is) and Incision inferius (Ii). Maximum values of unassisted movements were collected. The measurements were made by specialists who referred the patient for injection treatment and were aware of which preparation the patient was receiving.

### 2.6. Sample Size

The sample size was calculated using ClinCalc (version 2019.07.24; ClinCalc LLC, Arlington Heights, IL, USA) based on preliminary data on mandibular abduction for the 5-intervention injection treatment protocol [9,65]. The input abduction increase means were 4.5 mm (SD = 4.2 mm) in the HA group and 1.6 mm in the PRP group [9,65]. It was determined that for two independent study groups, a continuous primary endpoint, probability of type I error *α* = 0.05, probability of type II error 1 − *β* = 80%, and an equal enrollment ratio, it was necessary to include *n*_1_ = 33 patients in the study group and *n*_2_ = 33 patients in the control group.

### 2.7. Statistical Methods

The collected data were summarized in tables and visualized in charts. Means, standard deviations, mean differences, and standard errors were calculated. To determine statistical significance, *p*, a two-sided Student’s t-test was used, paired for calculations regarding changes in variable values in a single group of patients and homoscedastic for calculations between groups of patients. The effect size was estimated by calculating Cohen’s *d* for the confidence interval CI = 95%. Correlations were expressed with Pearson’s *r* coefficient.

Possible confounding factors were controlled for in three ways: (1) a control group was included to allow for a direct comparison of results and better isolation of the intervention effect from other potential variables, (2) matching based on the number and sex of patients was used to ensure comparability between the experimental and control groups, and (3) the absence of statistically significant differences in the baseline values of the variables between the groups was ensured.

Software used was as follows: (1) Google Workspace package (version 2024.05.31, Google LLC, Mountain View, CA, USA), (2) MedCalc software (version 22.023, MedCalc Software Ltd., Ostend, Belgium), and (3) Practical Meta-Analysis Effect Size Calculator application (version 2023.11.27, Campbell Collaboration, Philadelphia, PA, USA).

## 3. Results

### 3.1. Participant Flow

**Figure 2:**
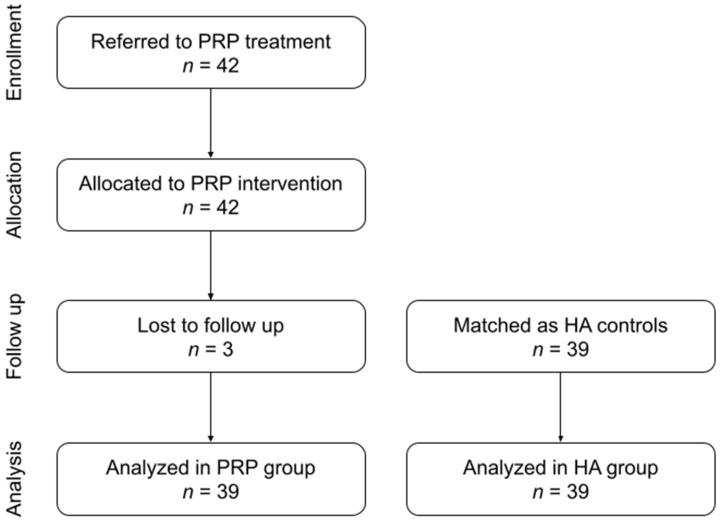
Flow diagram.

A total of 78 patients and 128 joints were included in the study, 39 patients and 69 joints in the PRP group and 39 patients and 59 joints in the HA group. Initially, the PRP group size was planned to be 42, but 3 patients withdrew after completion of the intervention but before the final physical examination (Figure 2). The HA group was matched to the PRP group in terms of the identical number of patients and sex structure, as well as similar baseline values of mandibular mobility.

### 3.2. Recruitment

Patients were recruited to the study group from 1 January 2021, to 31 December 2023. The control group included patients treated from the beginning of 2019. Patients in both groups were treated under individual schedules. The intervention was performed five times at intervals of 7 to 10 days, depending on organizational possibilities and the availability of patients. A medical history was collected, and a physical examination was performed immediately before and after the series of intra-articular administrations. Patients returned to their previous treatment immediately after completing the intervention protocol and collecting study outcomes.

### 3.3. Baseline Data and Numbers Analyzed

**Figure 3:**
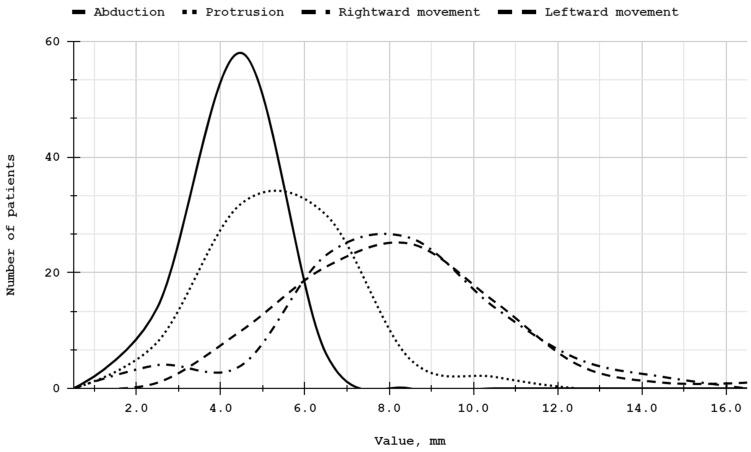
Distribution of baselines, quantified at 2 mm intervals. Abduction values scaled down by a factor of 10 for visualization consistency, with all other variables presented without scaling.

All patients enrolled in the study were Caucasian, aged 15 to 79 years. The male-to-female ratio was 0.11 in both patient groups. Table 2 presents further characteristics and baseline values of the mandibular mobility range variables. No statistically significant differences were observed between baseline values in the PRP and HA groups. The distributions were normal. Figure 3 presents the distributions of the initial values for individual mandibular movements in the entire sample. All patients were included in the analyses.

### 3.4. Outcomes and Estimation

**Figure 4:**
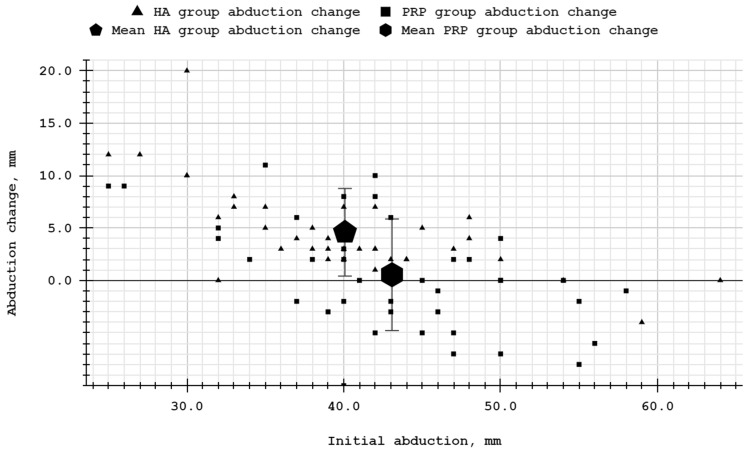
Mandibular abduction change compared to initial mandibular abduction.

Raw data for patient groups are available in Table A1 and Table A2. The mean mandibular mobility values for the PRP and HA groups and the differences between these groups are presented in Table 3. Mandibular mobility in all tested directions in the HA group changed statistically significantly compared to the baseline values. In the PRP group, the movement range did not change statistically considerably regarding each direction. Intergroup differences in abduction and protrusion were statistically significant, but not in lateral movements. Negative Cohen’s d values confirmed higher mandibular mobility improvements in the HA group than in the PRP group. The effect sizes were large and medium for abduction and protrusion, respectively. The values of the mandibular abduction change in individual patients are visualized in Figure 4. Data points in the charts are arranged from the top left to the bottom right, graphically representing the negative correlations between initial and increment values (*r* coefficients were −0.54 and −0.69 for PRP and HA, respectively).

### 3.5. Harms

There were 10 (26%) and 17 (44%) non-responders for TMJ pain in the PRP and HA groups, respectively. In a 30-year-old female treated with PRP bilaterally, the initially mild pain increased by 7 and 8 points on a 0–10 scale, in the right and left TMJ, respectively. In a 29-year-old female, the left joint deteriorated by 7 points, and the right joint improved by 1 point due to PRP treatment. The remaining non-respondents presented no change in pain intensity or worsening up to 3 points.

## 4. Discussion

TMJ injections are a minimally invasive surgical technique [69]. Open TMJ surgery is being replaced by arthroscopic surgery whenever possible, and blind TMJ injection is an approach derived from arthroscopy [70,71]. Intra-articular injections reduce the skin wound size and tissue trauma [69,70]. Although not allowing for surgery on tumors or fractures, they enable rinsing of the joint capsule, disruption of adhesions, removal of inflammatory mediators, supplementation of hyaluronic acid in synovial fluid (viscosupplementation), and administration of autografts or drugs [46,55,57,63,71]. In arthritis, disk displacement, and degenerative joint disease, injection therapy may effectively relieve articular pain and improve the mandibular range of motion [72]. Additional advantages of such treatment include a low risk of complications, low inconvenience of potential complications, ease of performing procedures, the possibility of outpatient treatment, short time of visits, quick results, the possibility of repeating the intervention in the case of a lack of response, and low price [9,58,64,65,68].

Among injection therapies, intra-articular administration is less invasive than arthrocentesis [65,68]. The superior compartment of the temporomandibular joint is more easily achieved, but there are scientific reports on the justification of administration to the inferior one [16,17]. The choice of the ideal injectable substance is the subject of current dispute among researchers [46,63]. It is already known that the administration of local anesthetics in this way is not justified [73]. Non-steroidal anti-inflammatory drugs used for this indication are being studied, and the current systematic review showed that individual drugs from this group should be treated individually [74].

Systematic reviews on HA intra-articular injections are contradictory [58,75,76]. However, it should be realized that it is difficult to evaluate therapies heterogeneous in terms of the use of arthrocentesis, the specific preparation, its concentration, volume, and number of administrations. This study was conducted under a protocol of a fixed number of five administrations. In everyday clinical practice, it seems reasonable to evade further interventions when the desired therapeutic effect is achieved [9].

It is presumed that in patients treated with HA, the composition of the synovial fluid improves by adding the lubricant. In the PRP group, there was neither improvement nor deterioration. This means that PRP does not affect joint lubrication. However, mechanical joint lubrication is not the only factor influencing mastication. Joint pain subsides with both HA and PRP treatment [9,38,65]. Its presence affects the range of painless mouth opening [65]. Therefore in this study, the maximum range of mandibular mobility was analyzed.

Recognizing the difference between HA and PRP in the presence or absence of the lubrication effect is important for more the appropriate qualification of patients for injection treatment. This is because not every TMD case presents limited mobility within TMJ [35,36]. PRP has anti-inflammatory and regenerative potential [22,77]. Nevertheless, it cannot be directly demonstrated in clinical studies with outcomes limited to a physical examination. Therefore, it should be emphasized that the better immediate effect presented in the HA group does not exclude a generally better treatment result in patients receiving PRP.

Regarding the above considerations, the appropriate placement of TMJ injectors on the therapeutic ladder is a current challenge. The selection of the study group from previously untreated patients and those for whom non-surgical treatment options have been exhausted takes two extremely different approaches, which may significantly affect the trial results. Our study recruited patients whose previous, less invasive treatment was ineffective.

The lack of statistically significant differences in the age structure and initial values of all examined mandibular mobility variables supports the stability of eligibility criteria despite non-simultaneous recruitment. The discrepancy between the study and control group results for all variables was unequivocal, and the effect size for the predominant one, abduction, was calculated to be large.

Despite the strengths mentioned, it must be emphasized that the trial was non-randomized and the control was non-concurrent. Both recipients and providers were aware of the assigned intervention during the trial. Therefore, the study outcomes are preliminary and ineligible for the highest evidence-level meta-analyses. The lack of a placebo group excluded the possibility of assessing the effect of needling alone or the deposition of a biologically inactive substance. It is known from other studies that the influence of the above-mentioned elements of the intervention cannot be neglected [78,79]. The lack of physical examinations between interventions precluded assessing the dynamics of mandibular mobility changes, which could be useful in investigating the most appropriate injection series length. Due to the study design, long-term therapeutic effects could not be determined.

Within the limits presented above, a five-intervention HA injection treatment protocol without arthrocentesis demonstrated a statistically significant improvement in mandibular mobility in patients with all types of TMJ pain except that attributed to subluxation [36]. Mandibular mobility increased statistically significantly in all tested directions following the proposed HA administration protocol. In the HA group, there was one case of a 4 mm deterioration and three cases of no change, which means a 90% response to treatment.

The overall rate of non-respondents for abduction in the PRP group was 22 (56%). In the study group, 17 patients showed a deterioration of no more than 10 mm, and in the remaining 5, no difference from the baseline value was observed. Combined with the lack of a statistically significant improvement from the initial values in all four tested directions of movements, this precludes the short-term effectiveness of the fivefold intra-articular PRP administration.

The superiority of HA over PRP was observed clinically but requires tissue-level interpretation. Intra-articular injections of PRP, although undoubtedly beneficial for suppressing inflammation, did not appear to produce a statistically significant increase in mandibular mobility in any of the movements tested. The predominance of HA results is probably due to the biochemical characteristics of this substance. It consists of repeating glucuronic acid and N-acetylglucosamine forming long polymer chains of several thousand to several million Daltons in molecular weight [80]. Patients of the study were provided with a 2.1 million Dalton HA. Such a high-weight HA creates viscous, elastic solutions that lubricate joints effectively [81]. Long HA chains form molecular networks retaining water and increasing the viscosity of synovial fluid. HA forms complexes with proteins and binds water, further improving the viscosity and elasticity of synovial fluid [82]. HA provides effective lubrication, shock absorption, and protection against mechanical damage in all physiological joint functions [10,80,82]. Inflammation, especially when chronic, reduces the HA content in synovial fluid. Therefore, HA supplementation restores the properties of the fluid and thus improves the functioning of the joints [40,83].

Moreover, HA exhibits mechanisms of action beyond the discussed properties. In addition to its known role in lubrication and shock absorption, HA has demonstrated an inhibitory effect on matrix metalloproteinase 13 (MMP13) and acts as a senomorphic agent, suggesting potential benefits in cellular aging and tissue regeneration [84]. Furthermore, HA has been shown to reduce vascular endothelial growth factor (VEGF) following injection, indicating a possible anti-inflammatory effect [85]. These findings suggest that HA may offer additional therapeutic advantages through its effects on inflammation and tissue remodeling [84,85].

## 5. Conclusions

The five-fold administration of hyaluronic acid into the temporomandibular joint cavities statistically significantly improved mandibular abduction, protrusion, and lateral mobility. Analogous treatment with platelet-rich plasma did not provide statistically significant improvement. These differences can be explained by the lubricating properties of hyaluronic acid. Therefore, in TMD cases manifesting mainly as a decrease in mandibular mobility, the intra-articular administration of HA, rather than PRP, seems to be more appropriate for symptomatic treatment.

## Figures and Tables

**Table 1:** Eligibility criteria.

	Criteria for Inclusion	Criteria for Exclusion
Diagnosis	ICOP 2020 diagnosis of TMJ pain [36]	ICOP 2020 detailed diagnosis of TMJ pain attributed to subluxation [36]
Medical history	A history of unsuccessful treatment of TMJ pain with less invasive methods, in particular, systemic pharmacotherapy, physiotherapy, or splint therapy	Previous TMJ arthroscopy or replacement
General condition	Ability to attend scheduled medical appointments	Active cancer, hematopoietic system disease, or bleeding diathesis
Local conditions	Possibility of discontinuing other TMJ pain treatment methods for the duration of the study	Disease of the skin or subcutaneous tissue of the preauricular area of the affected side, temporomandibular joint ankylosis, or temporomandibular joint prosthesis
Settings	Undergoing an initial and final examination and receiving a series of TMJ injections	Withdrawal from participation in the study at any stage

ICOP—International Classification of Orofacial Pain.

**Table 2:** Characteristics and baseline values.

	PRP + HA	SD	PRP	SD	HA	SD	MD	SE	*p*
Mean age	40.10	13.70	38.00	14.50	42.21	12.70	−4.21 †	3.09	0.18
Mandibular abduction	41.58	8.01	43.08	7.72	40.08	8.11	3.00 †	1.79	0.10
Mandibular protrusion	5.48	1.82	5.55	1.89	5.41	1.77	0.14 †	0.42	0.74
Rightward movement	8.06	2.43	8.38	2.41	7.74	2.44	0.64 †	0.55	0.25
Leftward movement	8.14	2.41	8.33	2.52	7.95	2.31	0.38 †	0.55	0.49

HA—hyaluronic acid patient group; MD—mean difference; *p*—statistical significance; PRP—platelet-rich plasma patient group; PRP + HA—all participants; SE—standard error; SD—standard deviation; †—no statistical significance.

**Table 3:** Outcomes in millimeters (mean values).

	PRP	SD	*p*	HA	SD	*p*	MD	SE	*p*	*d*	SE	CI from	CI to
Initial abduction	43.08	7.72		40.08	8.11		3.00 †	1.79	0.10				
Final abduction	43.62	6.58		44.67	6.04								
Abduction increase	0.54 †	5.32	0.53	4.59 *	4.18	0.00	−4.05 *	1.08	0.00	−0.85	0.24	−1.31	−0.38
Initial protrusion	5.55	1.89		5.41	1.77		0.14 †	0.42	0.74				
Final protrusion	6.32	1.66		7.15	1.44								
Protrusion increase	0.77 †	2.31	0.06	1.74 *	1.37	0.00	−0.97 *	0.43	0.03	−0.51	0.23	−0.96	−0.06
Initial rightward movement	8.38	2.41		7.74	2.44		0.64 †	0.55	0.25				
Final rightward movement	8.91	2.23		8.49	2.13								
Rightward movement increase	0.53 †	2.33	0.17	0.74 *	1.33	0.00	−0.21 †	0.43	0.63	−0.11	0.23	−0.55	0.33
Initial leftward movement	8.33	2.52		7.95	2.31		0.38 †	0.55	0.49				
Final leftward movement	8.99	2.32		8.90	1.82								
Leftward movement increase	0.65 †	2.27	0.08	0.95 *	1.26	0.00	−0.30 †	0.42	0.47	−0.16	0.23	−0.61	0.28

CI—confidence interval; *d*—Cohen’s *d*; HA—hyaluronic acid patient group; MD—mean difference; NA—not applicable; *p*—statistical significance; PRP—platelet-rich plasma patient group; SD—standard deviation; SE—standard error; *—statistical significance; †—no statistical significance

## Data Availability

The original contributions presented in the study are included in the article; further inquiries can be directed to the corresponding author.

## Appendix A

**Table A1:** PRP group raw data and change magnitudes.

Patient Number	Initial Abduction	Final Abduction	Abduction Change	Initial Protrusion	Final Protrusion	Protrusion Change	Initial Rightward Movement	Final Rightward Movement	Rightward Movement Change	Initial Leftward Movement	Final Leftward Movement	Leftward Movement Change
1	40.0	43.0	3.0	2.5	5.0	2.5	10.0	10.0	0.0	6.0	0.0	−6.0
2	42.0	50.0	8.0	5.0	5.0	0.0	10.0	7.0	−3.0	10.0	9.0	−1.0
3	40.0	30.0	−10.0	2.0	4.0	2.0	8.0	8.0	0.0	6.0	7.0	1.0
4	45.0	45.0	0.0	9.0	8.0	−1.0	11.0	8.0	−3.0	4.0	11.0	7.0
5	47.0	42.0	−5.0	5.0	4.0	−1.0	7.0	8.0	1.0	10.0	10.0	0.0
6	41.0	41.0	0.0	7.0	4.0	−3.0	12.0	9.0	−3.0	10.0	8.0	−2.0
7	56.0	50.0	−6.0	2.0	12.0	10.0	14.0	15.0	1.0	12.0	12.0	0.0
8	32.0	36.0	4.0	4.0	6.0	2.0	6.0	9.0	3.0	10.0	6.0	−4.0
9	42.0	37.0	−5.0	6.0	6.0	0.0	10.0	7.0	−3.0	7.0	10.0	3.0
10	50.0	54.0	4.0	6.0	7.0	1.0	12.0	13.0	1.0	12.0	13.0	1.0
11	42.0	52.0	10.0	5.0	6.0	1.0	7.0	8.0	1.0	7.0	9.0	2.0
12	37.0	35.0	−2.0	7.0	5.5	−1.5	10.0	8.0	−2.0	9.0	11.0	2.0
13	50.0	43.0	−7.0	6.0	5.0	−1.0	8.0	6.0	−2.0	6.0	6.0	0.0
14	40.0	42.0	2.0	5.0	6.0	1.0	8.0	8.0	0.0	10.0	10.0	0.0
15	46.0	45.0	−1.0	2.0	6.0	4.0	6.0	8.0	2.0	9.0	9.0	0.0
16	54.0	54.0	0.0	6.0	7.0	1.0	7.0	8.0	1.0	9.0	11.0	2.0
17	46.0	43.0	−3.0	5.0	5.0	0.0	7.0	12.0	5.0	14.0	12.0	−2.0
18	50.0	50.0	0.0	7.0	5.0	−2.0	10.0	11.0	1.0	13.0	13.0	0.0
19	25.0	34.0	9.0	5.0	5.0	0.0	8.0	9.0	1.0	9.0	8.0	−1.0
20	26.0	35.0	9.0	6.0	6.0	0.0	3.0	3.0	0.0	6.0	6.0	0.0
21	50.0	50.0	0.0	7.0	8.0	1.0	10.0	11.0	1.0	10.0	11.0	1.0
22	48.0	50.0	2.0	11.0	8.0	−3.0	6.0	9.0	3.0	7.0	10.0	3.0
23	43.0	40.0	−3.0	4.0	5.0	1.0	9.0	10.0	1.0	9.0	10.0	1.0
24	43.0	49.0	6.0	6.0	8.0	2.0	6.0	9.0	3.0	7.0	9.0	2.0
25	38.0	40.0	2.0	4.0	7.0	3.0	10.0	7.0	−3.0	8.0	10.0	2.0
26	35.0	46.0	11.0	3.0	9.0	6.0	6.0	9.0	3.0	5.0	8.0	3.0
27	32.0	37.0	5.0	3.0	3.0	0.0	3.0	6.0	3.0	5.0	8.0	3.0
28	55.0	47.0	−8.0	7.0	7.0	0.0	10.0	10.0	0.0	10.0	10.0	0.0
29	40.0	38.0	−2.0	7.0	7.0	0.0	13.0	7.0	−6.0	10.0	9.0	−1.0
30	34.0	36.0	2.0	6.0	6.0	0.0	8.0	10.0	2.0	7.0	9.0	2.0
31	47.0	40.0	−7.0	5.0	5.0	0.0	6.0	6.0	0.0	5.0	7.0	2.0
32	58.0	57.0	−1.0	7.0	7.0	0.0	10.0	13.0	3.0	9.0	9.0	0.0
33	43.0	41.0	−2.0	7.0	7.0	0.0	8.0	10.0	2.0	11.0	10.0	−1.0
34	47.0	49.0	2.0	7.0	6.0	−1.0	10.0	11.0	1.0	11.0	9.0	−2.0
35	45.0	40.0	−5.0	7.0	8.0	1.0	8.0	11.0	3.0	10.0	9.0	−1.0
36	39.0	36.0	−3.0	6.0	6.0	0.0	9.0	7.0	−2.0	5.0	6.0	1.0
37	55.0	53.0	−2.0	5.0	6.0	1.0	8.0	8.5	0.5	5.0	7.5	2.5
38	40.0	48.0	8.0	5.0	8.0	3.0	6.0	9.0	3.0	5.0	9.0	4.0
39	37.0	43.0	6.0	7.0	8.0	1.0	7.0	9.0	2.0	7.0	9.0	2.0

**Table A2:** HA group raw data and change magnitudes.

Patient Number	Initial Abduction	Final Abduction	Abduction Change	Initial Protrusion	Final Protrusion	Protrusion Change	Initial Rightward Movement	Final Rightward Movement	Rightward Movement Change	Initial Leftward Movement	Final Leftward Movement	Leftward Movement Change
1	33.0	41.0	8.0	5.0	7.0	2.0	12.0	12.0	0.0	8.0	10.0	2.0
2	38.0	43.0	5.0	5.0	7.0	2.0	10.0	11.0	1.0	5.0	8.0	3.0
3	50.0	52.0	2.0	5.0	7.0	2.0	11.0	11.0	0.0	8.0	10.0	2.0
4	59.0	55.0	−4.0	8.0	8.0	0.0	15.0	12.0	−3.0	16.0	13.0	−3.0
5	42.0	45.0	3.0	7.0	8.0	1.0	8.0	10.0	2.0	8.0	10.0	2.0
6	33.0	40.0	7.0	4.0	7.0	3.0	8.0	10.0	2.0	6.0	8.0	2.0
7	42.0	45.0	3.0	5.0	7.0	2.0	6.0	8.0	2.0	10.0	10.0	0.0
8	37.0	41.0	4.0	6.0	7.0	1.0	6.0	8.0	2.0	9.0	10.0	1.0
9	43.0	45.0	2.0	4.0	4.0	0.0	7.0	7.0	0.0	6.0	6.0	0.0
10	54.0	54.0	0.0	7.0	7.0	0.0	9.0	11.0	2.0	11.0	11.0	0.0
11	32.0	38.0	6.0	5.0	6.0	1.0	7.0	9.0	2.0	7.0	9.0	2.0
12	30.0	40.0	10.0	4.0	7.0	3.0	7.0	10.0	3.0	6.0	10.0	4.0
13	27.0	39.0	12.0	3.0	6.0	3.0	5.0	8.0	3.0	6.0	8.0	2.0
14	25.0	37.0	12.0	2.0	6.0	4.0	5.0	7.0	2.0	7.0	9.0	2.0
15	30.0	50.0	20.0	6.0	8.0	2.0	6.0	8.0	2.0	6.0	9.0	3.0
16	35.0	42.0	7.0	4.0	7.0	3.0	6.0	8.0	2.0	7.0	9.0	2.0
17	39.0	43.0	4.0	6.0	6.0	0.0	8.0	8.0	0.0	8.0	8.0	0.0
18	40.0	42.0	2.0	8.0	9.0	1.0	8.0	8.0	0.0	9.0	9.0	0.0
19	39.0	42.0	3.0	7.0	9.0	2.0	7.0	8.0	1.0	9.0	9.0	0.0
20	40.0	43.0	3.0	7.0	8.0	1.0	7.0	8.0	1.0	8.0	9.0	1.0
21	45.0	50.0	5.0	4.0	7.0	3.0	6.0	8.0	2.0	8.0	8.0	0.0
22	48.0	54.0	6.0	4.0	8.0	4.0	3.0	3.0	0.0	7.0	8.0	1.0
23	48.0	52.0	4.0	6.0	7.0	1.0	9.0	9.0	0.0	9.0	10.0	1.0
24	64.0	64.0	0.0	7.0	7.0	0.0	12.0	12.0	0.0	12.0	12.0	0.0
25	30.0	40.0	10.0	5.0	5.0	0.0	5.0	8.0	3.0	5.0	8.0	3.0
26	40.0	42.0	2.0	8.0	8.0	0.0	9.0	9.0	0.0	8.0	9.0	1.0
27	40.0	45.0	5.0	7.0	8.0	1.0	8.0	8.0	0.0	8.0	8.0	0.0
28	40.0	47.0	7.0	8.0	8.0	0.0	9.0	10.0	1.0	10.0	10.0	0.0
29	47.0	50.0	3.0	10.0	10.0	0.0	9.0	11.0	2.0	11.0	12.0	1.0
30	39.0	41.0	2.0	7.0	9.0	2.0	8.0	8.0	0.0	7.0	8.0	1.0
31	42.0	43.0	1.0	4.0	8.0	4.0	6.0	6.0	0.0	7.0	8.0	1.0
32	38.0	41.0	3.0	4.0	7.0	3.0	8.0	8.0	0.0	8.0	8.0	0.0
33	32.0	32.0	0.0	2.0	2.0	0.0	3.0	2.0	−1.0	2.0	2.0	0.0
34	36.0	39.0	3.0	4.0	6.0	2.0	4.0	5.0	1.0	5.0	6.0	1.0
35	41.0	44.0	3.0	5.0	9.0	4.0	9.0	8.0	−1.0	8.0	9.0	1.0
36	44.0	46.0	2.0	5.0	8.0	3.0	8.0	8.0	0.0	8.0	8.0	0.0
37	44.0	46.0	2.0	4.0	8.0	4.0	9.0	8.0	−1.0	9.0	9.0	0.0
38	42.0	49.0	7.0	5.0	7.0	2.0	10.0	9.0	−1.0	9.0	10.0	1.0
39	35.0	40.0	5.0	4.0	6.0	2.0	9.0	9.0	0.0	9.0	9.0	0.0
